# Does the air condition system in busses spread allergic fungi into driver space?

**DOI:** 10.1007/s11356-017-0830-4

**Published:** 2017-12-05

**Authors:** Małgorzata Sowiak, Anna Kozajda, Karolina Jeżak, Irena Szadkowska-Stańczyk

**Affiliations:** Nofer Institute of Occupational Medicine, 8 Teresy Str, 91-348 Łódź, Poland

**Keywords:** Buses, Cars, Air-condition system, Ventilation system, Occupational exposure, Drivers, Mold, Fungi

## Abstract

The aim of this study was to establish whether the air-conditioning system in buses constitutes an additional source of indoor air contamination with fungi, and whether or not the fungi concentration depends on the period from the last disinfection of the system, combined with replacement of the cabin dust particle filter. The air samples to fungi analysis using impact method were taken in 30 buses (20 with an air-conditioning system, ACS; 10 with a ventilation system, VS) in two series: 1 and 22 weeks after cabin filter replacement and disinfection of the air-conditioning system. During one test in each bus were taken two samples: before the air-conditioning or ventilation system switched on and 6 min after operating of these systems. The atmospheric air was the external background (EB). After 1 week of use of the system, the fungi concentrations before starting of the ACS and VS system were 527.8 and 1053.0 cfu/m^3^, respectively, and after 22 weeks the concentrations were 351.9 and 1069.6 cfu/m^3^, respectively. While in the sample after 6 min of ACS and VS system operating, the fungi concentration after 1 week of use was 127.6 and 233.7 cfu/m^3^, respectively, and after 22 weeks it was 113.3 and 324.9 cfu/m^3^, respectively. Results do not provide strong evidence that air-conditioning system is an additional source of indoor air contamination with fungi. A longer operation of the system promoted increase of fungi concentration in air-conditioned buses only.

## Introduction

The quality of indoor air is very important for humans’ health and well-being, because they spend more and more time in closed environments (even 90%) due to the development of civilization (Norback 2009). The factors which affect the quality of indoor air include airborne microorganisms, especially fungi, which co-create the bioaerosol. Depending on their level and qualitative composition, bioaerosols may contribute to the occurrence of various symptoms and diseases in humans (Srikanth et al. 2008). It has been indicated that exposure to fungal aerosol in the indoor air causes—among other—asthma and the sick building syndrome (SBS) (Wiszniewska et al. 2004; Piecková 2012; Eduard 2009; Bolashikov and Melikov 2009). Important factors which modify bioaerosol in the indoor air are ventilation and air-conditioning systems (Prussin and Marr 2015). The studies on the impact of those systems on the quality of indoor air indicated their purifying effects at longer and more frequent work of the air-conditioning system. However, with badly designed or maintained system of ventilation or air-conditioning, it becomes an additional source of fungal aerosol introduced into indoor air (Hamada and Fujita 2002). These studies, however, were related mainly to residential buildings (Garrison et al. 1993; Kemp et al. 2003), hospitals (Perdelli et al. 2006; Crimi et al. 2006), and offices (Graudenz et al. 2004, Bródka et al. 2012). An equally important indoor air environment is that of the cabin of vehicles which are more and more often conventionally equipped with the air-conditioning system. So far, the studies of indoor air quality in vehicles were focused on estimation of passengers and drivers’ exposure to particulate matters (PM) (Riediker et al. 2003; Zeldin et al. 2006; Knibbs et al. 2010), carbonyl compounds (Pang and Mu 2007), and volatile organic compounds (VOC) (Riediker et al. 2003; Parra et al. 2008; Janicka et al. 2011), and to a lower extent—to vegetable pollen (Hugg et al. 2007). There are few studies describing vehicle users’ exposure to bioaerosols, especially fungal aerosol (Lee and Jo 2005; Li et al. 2013; Vonberg et al. 2010). However, the few existing reports point to an important role of air-conditioning system in the development of indoor air quality in cars. It has been indicated that under the conditions of high humidity and availability of substrates, the fungi may growth on air filters surface and other elements in air-conditioning systems. For their growth, they use the humidity arising in the steamer during the air-conditioning system functioning and organic compounds contained in the deposited dust coming from the atmospheric air (Maus et al. 2001). These microorganisms may penetrate into indoor air, causing secondary air pollution in vehicles and contribute to the occurrence of diseases, including allergy in drivers and travelers (Vonberg et al. 2010). They may also be a source of volatile organic compounds inside the cars (Rose et al. 2000). Determination of exposure to fungal aerosol coming from the air-conditioning system in vehicles is particularly important in the case of professional drivers who spend a lot of time inside the cars. This group involves city bus drivers who spend on average about 8 h in closed cabins of vehicles, with the switched on air-conditioning system in summer season.

The aim of this study was to establish whether the air-conditioning system in buses constitutes an additional source of indoor air contamination with fungi (especially allergenic genera), and whether or not the fungi concentration depends on the period from the last disinfection of the system, combined with replacement of the cabin dust particle filter.

## Material and methods

### Air sample collection and analysis

For the purpose of the results, presentations were used in the abbreviations listed below:

- ACS—air conditioning system;
- VS—ventilation system;
- BACS—buses with air conditioning system;
- BVS—buses with ventilation system

The study examined 30 buses of the same type, operated by an urban public transport company. All buses were provided with a usual ventilation system (VS) comprising air inlet and outlet channels, vents/supply air outlets supplying air to various locations in the cab, the blow/exhaust fan to ensure air circulation in the cab, and the cabin air filter (CAF) to stop dust pollution placed in the intake passage to the vents. The vehicles under study were divided into two groups: buses only equipped with a ventilation system (BVS, *N* = 10) and buses with installed additional air-conditioning system (BACS, *N* = 20) with the possibility of an independent activation of air-conditioning in both the cab and in the passenger space. Each bus was tested by us twice in the same year: in May and September. The periods of the testing had been chosen so that the time interval between the successive tests included the warmest months in Polish climatic conditions, i.e., the period of intensive use of air-conditioning systems in vehicles. The first test was conducted 1 week after cabin filter replacement in buses; in the air-conditioned vehicles, the replacement procedure was combined with simultaneous disinfection of the air-conditioning system. The other test was performed after 22 weeks of use of a new filter and—in air-conditioned buses—disinfected air-conditioning system. Technical data on the buses and exact dates of replacement of the cabin air filter and disinfection of the air-conditioning system in each vehicle were obtained from the company that owns the buses. During each test, two air samples were collected to enable quantitative and qualitative analysis of airborne molds. The samples were collected inside the driver’s cab with windows and doors closed. Before starting the sampling procedure in each vehicle, air circulation in the cabin was set to on, the intensity of air flow to the cabin was set at 75%, and the temperature of the blowing air was set to the lowest attainable level, with the air flow directed only at the breathing zone of the driver with the remaining supply nozzles closed. The first sample (P1) was collected before the ACS or VS switched on, while the second sample (P2) was collected after 6 min of the operation of the ACS (in buses with air-conditioning system) or the VS (in buses without air-conditioning system). Simultaneously, for each, sampling series were taken outdoor air samples as the external background of the study (EB).

The air samples were collected at the zone approximately corresponding to the driver breathing zone (about 0.5 m from vents/supply air outlets located next to the steering wheel) using a portable air sampler for agar plates (Burkard Manufacturing Co Ltd., England) with a flow rate of 20 l/min. Before each sampling, the inside of the sampler was disinfected with 70% isopropyl alcohol. Air samples were collected directly on plates with malt extract agar (soy peptone—3 g/l, agar—15 g/l, malt extract—30 g/l (BTL Sp. z o.o., Poland)) supplemented with streptomycin sulfate (130 mg/l (Sigma-Aldrich Chemie GmbH, Germany)) and chloramphenicol (50 mg/l (PPH Galfarm Sp. z o.o., Poland)). The sampling period of each sample was 5 min. Additionally, to determine the “background” concentrations for airborne molds on the outside of each vehicle covered by the study at the height of 1.0–1.2 m over the ground level, an atmospheric air sample (EB) was taken in the same way. All samples were collected in two repetitions.

After sampling, agar plates were incubated at 25 ± 1 °C for 7 days. The colonies which appeared on the plates were enumerated. The “positive-hole method” was applied to samples for corrections of microbial coincidence (Peto and Powell 1970). All concentrations of total culturable airborne molds were expressed as the number of colony-forming units on the culture medium per cubic meter of the examined air (cfu/m^3^). The detection limit of the sampling procedure was 10 cfu/m^3^. The concentrations for samples in which there was no growth (the concentrations were below the detection limits) were defined as 5 cfu/m^3^ (half of detection limit) for the purposes of analysis. The results were averaged for the repetitions.

The colonies on the plates were analyzed for belonging to four genera of molds most allergenic to humans: *Aspergillus*, *Penicillium*, *Cladosporium*, *Alternaria*, and pathogenic species from *Fumigati section*. Molds were identified on the basis of colonial morphology on diagnostic media and on microscopic morphology by keys to identification available in the literature—as previously described (Fisher and Cook 1998; Pitt 2000; Flannigan et al. 2001; Klich 2002; Larone 2002; Samson et al. 2004). According to the International Code of Nomenclature for fungi and based on knowledge about presence of closely related species (cryptic species), among *Aspergillus* genera in our study, *Aspergillus* classified all species from four subgenera and all sections including unreferenced species (Gautier et al. 2016; Houbraken et al. 2014). Fungi that cannot be distinguished based on morphological features from *Aspergillus fumigatus* species were determined as *Aspergillus section*. The concentrations of specified mold genus/section per cubic meter of air have been estimated from their percentages in the total concentration of airborne molds, which had been calculated taking into account the correction of microbial coincidence.

During indoor (only P1) and outdoor bioaerosol sampling at the same point, the basic parameters of microclimate, such as temperature and relative humidity of air were measured. The measurements were carried out using the microclimate multifunction meter Testo 435-2 (Testo AG, Germany) during 5 min. The values of individual parameters were read out every minute, then the result was averaged for a given measurement point.

### Statistical analysis

The total concentrations of airborne culturable fungi inside and outside the buses were characterized by using the geometric mean (GM), geometric standard deviation (GSD), and the range of the observed values (MIN–MAX). The concentrations of the mold genus/section and the values of indoor and outdoor microclimate parameters were characterized by using arithmetic mean (AM) standard deviation (SD) and the range of the observed values.

In order to determine the change in the total concentration of airborne molds inside the buses due to the activity of the ACS or VS, the ratio of concentration determined after the activation of the system to that obtained prior to the activation of the system (P2/P1 ratio) was calculated for each bus and the results were expressed as percent values. The ratio could not be calculated for concentrations of some mold genera/section, because their concentrations in many samples collected prior to the activation of the system (P1) was 0 cfu/m^3^.

In order to determine the change in the concentration of airborne mold *Aspergillus*, *Penicillium*, *Cladosporium*, *Alternaria* genera, and *Fumigati* section inside the buses resulting from the operation of the ACS or VS, the incidence of increased concentration of these fungi after the activation of the system compared to their concentration prior to its activation was calculated. The obtained incidences were expressed as percent values.

The Kolmogorov-Smirnow and Shapiro-Wilk tests were used to ascertain the normality of the continuous distribution of P2/P1 indicator values. Because the values of P2/P1 ratio were not normally distributed, the non-parametric statistics were used for hypothesis testing.

The significance of the impact of the air-conditioning system operation in the buses on the values of the P2/P1 ratio was estimated by the Mann-Whitney (U) test. The differences between values of the indicator in air-conditioned buses and in buses without air-conditioning system were evaluated twice: after 1 and after 22 weeks since replacing the cabin filter and—in the air-conditioned buses—since the simultaneous disinfection of the ACS.

The Wilcoxon’s rank sum test was applied to compare the differences between the paired values of P2/P1 ratio obtained for the same buses after 1 and after 22 weeks of use of new cabin filter and—in the air-conditioned buses—after 1 and after 22 weeks of use of the ACS after disinfection. These differences were evaluated separately for the BACS and for the BVS.

The significance of the influence of the ACS in buses on the increase of the concentration of specific airborne mold genera/section after the starting of this system was estimated by non-parametric Fischer’s exact test for qualitative variables. The differences between incidences in BACS and in BVS were evaluated twice: after 1 and after 22 weeks from the moment of replacing the cabin filter and—in air-conditioned buses—from the moment of the ACS disinfection.

The non-parametric McNemar’s test for qualitative variables was applied to compare the differences between the paired values of incidence of increase in the mold genus/section concentration after the startup of the system obtained for the same buses after 1 and after 22 weeks of use of new cabin filter and—in air-conditioned buses—also after the disinfected ACS. These differences were evaluated separately for the BACS and for the BVS.

All statistical analyses were performed with STATISTICA v. 7.1 software package (StatSoft Polska Sp. z o.o., Poland). *P* value of 0.05 was considered as the level of significance.

## Results

Analysis of microclimatic conditions prevailing inside the vehicles before starting the air-conditioning or ventilation system showed that the arithmetic mean air temperature in the air-conditioned buses and in those without air-conditioning system was similar. In both types of the tested buses, the average temperature inside the vehicle did not differ remarkably from the average outside air temperature. In turn, the arithmetic mean relative humidity of the inside air before starting of the air-conditioning or ventilation system after 1 week of use of the air-conditioned buses was higher than in the non-air-conditioned buses. The average relative humidity values of the air inside the driver compartment of the tested vehicles were higher than the corresponding values of the outside atmospheric air, with the exception of non-air-conditioned buses examined after 22 weeks of use of the ventilation system, where the values were at a level similar to that of the outside atmospheric air. Detailed data concerned microclimate conditions are showed in Table 1. (Table 1).

**Table 1:** Microclimatic parameters in the indoor air in buses, air in buses with and without air-conditioning system, and in outdoor air (external background) regarding to period of the operation of the system (*N* = 30)

Type of buses	Period of system operation^a^ (weeks)	Temperature (°C)		Relative humidity (%)	
		P1	EB	P1	EB
		AM (SD) MIN-MAX	AM (SD) MIN-MAX	AM (SD) MIN-MAX	AM (SD) MIN-MAX
With air-conditioning system (*N* = 20)	1	21.0 (5.6) 13.8–29.2	21.1 (8.2) 13.2–33.5	61.8 (16.6) 37.5–84.5	57.8 (25.7) 35.6–88.4
	22	17.6 (3.3) 12.2–23.6	18.2 (2.3) 16.0–20.4	60.8 (8.8) 48.0–74.3	55.0 (10.0) 45.2–64.7
Without air-conditioning system (*N* = 10)	1	22.5 (6.4) 15.6–34.7	24.7 (7.6) 18.8–33.5	55.1 (7.0) 43.8–66.8	42.4 (5.9) 35.6–47.0
	22	14.5 (1.5) 13.2–17.8	11.7^b^	74.1 (6.6) 63.2–83.0	75.5^b^

*N* samples number, *P1* indoor air samples befor turning on air-conditioning system/ventilation system, *EB* external background (atmospheric air), *M* arithmetic mean, *SD* standard deviation, *MIN* minimum value, *MAX* maximum value

^a^For the buses with air-conditioning system: period of operation form the last filter replacement and disinfection; for the buses without air-conditioning system: period of operation from the last filter replacement

^b^Single sample microclimatic parameter in the outdoor air (the average value from the day of sampling)

Table 2 shows the geometric mean concentrations of total culturable molds found in the inside air of buses before and after starting of the air-conditioning or ventilation system, taking into account the period of operation of the system and the average concentrations of molds in the outside air. It was found that, regardless of the period of operation of the air-conditioning or ventilation system, before starting of the system, mean mold concentrations in air-conditioned buses were lower than in the buses not provided with air-conditioning system. After 1 week of use of the system, the corresponding concentrations were 527.8 and 1053.0 cfu/m^3^, respectively, and after 22 weeks the concentration values were 351.9 and 1069.6 cfu/m^3^, respectively. However, a similar relationship was observed also for the corresponding concentrations of the mold in the outside air. In both types of the examined buses, the average concentrations inside the vehicle before starting of the systems were also at least twice lower than the corresponding mean concentration of mold in the outside air. After system starting, regardless of the period of its operation, the mean concentrations of molds in the inside air of the buses were seen to decrease, while in the air-conditioned buses the concentrations were usually lower than in the buses without air-conditioning system. After 1 week of use of the system, the concentration was 127.6 and 233.7 cfu/m^3^, respectively, and after 22 weeks it was 113.3 and 324.9 cfu/m^3^, respectively.

**Table 2:** Concentration of total molds in the indoor air in buses with and without air-conditioning system and in the outdoor air (external background) regarding to period of the operation of the system (*N* = 30)

Type of buses	Period of system operation (weeks)	Total molds (cfu/m^3^)		
		P1	P2	EB
		GM (GSD) MIN-MAX	GM (GSD) MIN-MAX	GM (GSD) MIN-MAX
With air-conditioning system (*N* = 20)	1	527.8 (1.8) 105.0–1386.0	127.6 (3.0) 5.0–616.0	1229.5 (1.6) 892.0–2661.0
	22	351.9 (2.2) 41.0–1386.0	113.3 (1.9) 30.0–580.0	732.5 (1.4) 511.0–1050.0
Without air-conditioning system (*N* = 10)	1	1053.0 (1.3) 654.0–1561.0	233.7 (2.1) 73.0–734.0	2579.8 (1.0) 2527.0–2661.0
	22	1069.6 (1.5) 654.0–2303.0	324.9 (1.5) 174.0–635.0	2121.0^b^

*N* samples number, *P1* indoor air samples before turning on air-conditioning system/ventilation system, *P2* indoor air samples after 6 min operation of the air-conditioning system/ventilation system, *EB* external background (atmospheric air), *GM* geometric mean, *GSD* geometric standard deviation, *MIN* minimum value, *MAX* maximum value

^a^For the buses with air-conditioning system: period of operation form the last filter replacement and disinfection; for the buses without air-conditioning system: period of operation from the last filter replacement

^b^Single sample microclimatic parameter in the outdoor air (the average value from the day of sampling)

Table 3 summarizes mean values of the percentage ratio P2/P1, which represents the change in absolute units of the total concentration of mold in the air inside the buses after 6 min since starting of the air-conditioning or the ventilation systems relative to the concentration before system starting, taking into account the period of operation of the system.

**Table 3:** Percentage ratio (P2/P1) indicated changes in concentrations of total molds in the indoor air of buses with and without air-conditioning system after 6 min operation of the air-conditioning system relative to the state before turning on regarding to the period of operation both systems (*N* = 30)

Type of buses	P2/P1 (%)		*p*
	Period of system operation^a^ (weeks)		
	1	22	
	GM MIN-MAX	GM MIN-MAX	
With air-conditioning system (*N* = 20)	24.2 0.8–132.4	32.2 10.9–178.0	> 0.05
Without air-conditioning system (*N* = 10)	22.2 4.7–63.6	30.4 15.5–56.6	> 0.05
*p*	> 0.05	> 0.05	

*N* samples number, *P1* indoor air samples before turning on air-conditioning system/ventilation system, *P2* indoor air samples after a 6-min operation of the air-conditioning system/ventilation system, *EB* external background (atmospheric air), *GM* geometric mean, *GSD* geometric standard deviation, *MIN* minimum value, *MAX* maximum value

*p* level of statistical significance (*p* < 0.05)

^a^For the buses with air-conditioning system: period of operation form the last filter replacement and disinfection; for the buses without air-conditioning system: period of operation from the last filter replacement

In both types of vehicles tested, regardless of system operation time period, after starting of the system, a decline by ca. 68–78% was observed in air pollution by fungal microflora. In the air-conditioned buses, after 6 min of operation of the system, slightly more fungi remained in the inside air than in buses without air-conditioning system, and after 1 week of use of the system, fungal microflora pollution was 24.2 and 22.2% of the concentration before system starting, while after 22 weeks the corresponding percentages were 32.2 and 30.4%, respectively. These differences, however, were not statistically significant (*p* > 0.05). Taking into account the period of operation of the system, in both types of vehicles after 22 weeks of use of the system, a smaller average decrease in the concentration of mold was observed after system starting (on average by 68–70%) than after 1 week of operation (on average by 76–78%). Those differences were not statistically significant (*p* > 0.05). Despite the decline in average mold concentration observed after system starting in both types of vehicles, it is worth noting that the maximum value of mold concentration gradient P2/P1 for the air-conditioned buses was as high as 132.4% after 1 week of use of the system, and 178.0% after 22 weeks of use. This means that in some vehicles there was an increase in the concentration of mold after starting of the air-conditioning system relative to the pre-starting concentration, while after prolonged use of the system the observed maximum increase of the concentration was higher. Meanwhile, regardless of the period of use of the ventilation system, the maximum value of the P2/P1 index for non-air-conditioned buses never exceeded 100%.

Table 4 shows the arithmetic mean concentration of allergenic molds of the genera *Aspergillus*, *Penicillium*, *Cladosporium*, and *Alternaria* and the *Fumigati section* in the inside air of buses before and after starting of the air-conditioning or ventilation system, taking into account the period of use of the system, and the corresponding mean concentrations of these molds in the outside air.

**Table 4:** Concentration of molds belonging to *Aspergillus*, *Penicillium*, *Cladosporium*, and *Alernaria* genera, and *Fumigati section* in the indoor air in the buses with and without air-conditions system regarding to period of operation both systems^a^ (*N* = 30)

Type of buses	Period of system operation^a^ (weeks)	Concentrations of molds (cfu/m^3^)		
		P1	P2	TZ
		AM(SD) MIN-MAX	AM(SD) MIN-MAX	AM(SD) MIN-MAX
*Aspergillus* spp.				
With air-conditioning system (*N* = 20)	1	2.8 (6.0) 0.0–18.5	1.2 (3.7) 0.0–13.4	0.0
	22	10.5 (15.6) 0.0–59.2	0.5 (2.3) 0.0–10.4	0.0
Without air-conditioning system (*N* = 10)	1	5.4 (12.7) 0.0–38.8	3.6 (7.8) 0.0–21.7	16.5 (14.2) 0.0–27.5
	22	10.6 (14.0) 0.0–43.6	2.5 (8.0) 0.0–25.2	24.1^b^
*Fumigati* section				
With air-conditioning system (*N* = 20)	1	0.9 (4,1) 0.0–18.5	0.5 (2.3) 0.0–10.4	0.0
	22	1.3 (4.0) 0.0–14.8	0.0	0.0
Without air-conditioning system (*N* = 10)	1	5.4 (12.7) 0.0–38.8	3.6 (7.8) 0.0–21.7	16.5 (14.2) 0.0–27.5
	22	10.6 (14.0) 0.0–43.6	0,0	24.1^b^
*Penicillium* spp.				
With air-conditioning system (*N* = 20)	1	34.1 (35.6) 0.0–147.8	7.5 (10.8) 0.0–32.1	7.2 (12.7) 0.0–28.6
	22	34.1 (35.5) 0.0–121.2	9.9 (22.0) 0.0–97.8	12.8 (13.1) 0.0–25.6
Without air-conditioning system (*N* = 10)	1	47.6 (52.2) 0.0–155.3	10.4 (18.2) 0.0–56.5	44.4 (13,6) 28.6–54.9
	22	20.1 (21.1) 0.0–54.6	4.9 (10.5) 0.0–27.0	0.00^b^
*Cladosporium* spp.				
With air-conditioning system (*N* = 20)	1	262.2 (266.6) 0.0–1016.4	48.5 (39.1) 0.0–159.1	875.0 (768.8) 287.3–2146.0
	22	213.3 (224.9) 10.3–997.9	59.1 (66.3) 0.0–303.2	431.7 (220.1) 217.2–646.2
Without air-conditioning system (*N* = 10)	1	777.7 (222.6) 531.4–1242.3	201.7 (169.9) 52.2–578.7	2176.8 (26.6) 2146.0–2197.4
	22	248.9 (200.1) 103.6–785.6	49.9 (27.8) 0.0–81.7	1132.8^b^
*Alternaria* spp.				
With air-conditioning system (*N* = 20)	1	8.6 (11.6) 0.0–37.0	0.0	34.2 (48.1) 0.0–114.5
	22	22.3 (35.5) 0.0–147.8	4.3 (12,5) 0.0–51.9	85.5 (61.5) 25.6–145.4
Without air-conditioning system (*N* = 10)	1	16.5 (19.5) 0.0–58.2	18.7 (59.0) 0.0–186.7	62.3 (44.9) 27.5–114.5
	22	1.4 (4.3) 0.0–13.6	0.0	0.0^b^

*N* samples number, *P1* indoor air samples before turning on air-conditioning system/ventilation system, *P2* indoor air samples after a 6-min operation of the air-conditioning system/ventilation system, *EB* external background (atmospheric air), *AM* arithmetic mean, *SD* standard deviation, *MIN* minimum value, *MAX* maximum value

^a^For the buses with air-conditioning system: period of operation form the last filter replacement and disinfection; for the buses without air-conditioning system: period of operation from the last filter replacement

^b^Single sample microclimatic parameter in the outdoor air (the average value from the day of sampling)

Regardless of the period of operation of the air-conditioning or ventilation system, before its starting, the highest mean concentration in the inside air of both types of buses was observed for mold of the genera *Cladosporium*, followed in the descending order by *Penicillium* and *Alternaria*, and the lowest for *Aspergillus*. A similar trend was observed after starting of the system. In the outside atmospheric air serving for the background in this study, *Cladosporium* and *Alternaria* genera were dominant, while the levels of the genera *Penicillium* and *Aspergillus* were lower.

Detailed analysis of the concentrations of these four genera of molds showed that, regardless of the period of operation of the air-conditioning or ventilation system, prior to system starting in air-conditioned buses, lower mean levels of all tested genera of fungi were observed than in the buses without air-conditioning system. Exceptions included mean concentrations of *Penicillium* and *Alternaria* genera observed before system starting after 22 weeks of its use in air-conditioned buses, higher than mean concentrations recorded in non-air-conditioned buses after the same period of operation of the system. However, the same relationships as those shown in the above analysis of the internal concentrations were observed also for the corresponding mean concentrations of the individual genus of molds in the external background.

After starting of the system, regardless of the period of its use, there were decreases in the mean concentration of all tested genera of molds in the inside air of both types of buses, except for buses without air-conditioning system examined after 1 week of use of the system, where the average concentration of the genus *Alternaria* mold was slightly higher. In other instances, at least a 5-fold decrease in the mean concentration inside the vehicle was recorded for the *Alternaria* mold, at least 3-fold reduction for the *Cladosporium* and *Penicillium* molds, and at least 1.5-fold decrease for the *Aspergillus* mold. After 1 week of use of the system, soon after its starting, a deeper reduction of average mold concentration could be observed in the air-conditioned (compared to non-air-conditioned) buses for the *Cladosporium* and Aspergillus molds; the reduction was similar for the *Penicillium* mold, while the *Alternaria* mold was completely eliminated from the air. After 22 weeks of use of the system, soon after its starting, a lesser reduction of mean mold concentration could be observed in the air-conditioned (compared to non-air-conditioned) buses for all examined molds with the exception of the genus *Aspergillus*. Considering the period of operation of the system, after 22 weeks of its use, soon after its starting, the reduction in the average concentration of the *Penicillium* mold in both types of vehicles was smaller than after 1 week of operation. For the *Alternaria* and *Cladosporium* molds, such relationship was observed only in the air-conditioned buses. No lesser decrease in the average concentrations of the *Aspergillus* mold attributable to the starting of the system was observed after the longer period of operation of the system.

The *Fumiagti section* was found in the indoor air of some of the tested vehicles before staring of the air-conditioning or ventilation system. Its mean concentration was lower in the air-conditioned buses than in those without air-conditioning system, regardless of the period of system operation. However, a similar relationship was observed also for the concentrations corresponding to mean levels of that species in the outside background. At the same time, the average inside concentration of that mold before starting of the system in air-conditioned buses was at least one order of magnitude higher than in the atmospheric air, which was free of that species. In the buses without air-conditioning, it was at least a 2-fold lower than in the background. After starting of the system, regardless of the period of its use, the average concentration of *Fumigati section* in the inside air of both types of buses was seen to decrease, and after prolonged use of the system the decrease was more evident.

Table 5 shows the frequency of higher concentrations of the studied molds and *Fumigati section* in the samples of the indoor air collected after 6 min since starting of the air-conditioning or ventilation system, compared to the state before the starting. The table takes also into account the period of use of the system.

**Table 5:** The incidence of higher levels of molds belonging to *Aspergillus*, *Penicillium*, *Cladosporium*, and *Alternaria* genera and *Fumigati section* after a 6-min operation of the air-conditioning system relative to the state before turning on regarding to the period of operation both systems (*N* = 30)

Type of buses	Period of system operation^a^ (weeks)				*p*
	1		22		
	*n*	%	*n*	%	
*Aspergillus* spp*.*					
With air-conditioning system (*N* = 20)	2	10	0	0	> 0.05
Without air-conditioning system (*N* = 10)	1	10	1	10	> 0.05
*p*	> 0.05		> 0.05		
*Fumigati* section					
With air-conditioning system (*N* = 20)	1	5	0	0	> 0.05
Without air-conditioning system (*N* = 10)	1	10	0	0	> 0.05
*p*	> 0.05		> 0.05		
*Penicillium* spp.					
With air-conditioning system (*N* = 20)	3	15	3	15	> 0.05
Without air-conditioning system (*N* = 10)	2	20	0	0	> 0.05
*p*	> 0.05		> 0.05		
*Cladosporium* spp*.*					
With air-conditioning system (*N* = 20)	5	25	2	10	> 0.05
Without air-conditioning system (*N* = 10)	0	0	0	0	> 0.05
*p*	> 0.05		> 0.05		
*Alternaria* spp*.*					
With air-conditioning system (*N* = 20)	0	0	2	10	> 0.05
Without air-conditioning system (*N* = 10)	1	10	0	0	> 0.05
*p*	> 0.05		> 0.05		

*N* samples number, *n* number of samples with increased concentration of molds after a 6-min operation of air-conditioning system/ventilation system relative to the state befor turning on both system, *%* percentage contribution of samples with increased concentration of molds after a 6-min operation of air-conditioning system/ventilation system relative to the state before turning on both system

*p* level of statistical significance (*p* < 0.05

^a^For the buses with air-conditioning system: period of operation form the last filter replacement and disinfection; for the buses without air-conditioning system: period of operation from the last filter replacement

For both types of tested vehicles, the analysis showed low (0–25%) incidence of increased concentrations of the tested mold genera after starting of the system, regardless of the period of its operation. In the air-conditioned buses, after 1 week of using the system, a 25% of samples with higher mold concentration, compared to non-air-conditioned buses, incidence of higher levels after system staring was noted only for mold of the genus *Cladosporium*. For the *Aspergillus* mold, the incidence was 10% for both types of buses, and for the mold of the genera *Penicillium* and *Alternaria* it was higher in vehicles without air-conditioning system. After 22 weeks of operation of the system, soon after its starting, the frequency of higher mold concentrations in air-conditioned buses vs. non-air-conditioned ones was higher for all examined genera of molds with the exception of the genus *Aspergillus*. For the latter mold, higher percentage of vehicles with higher levels after starting of the system was observed among non-air-conditioned buses. However, the differences between the air-conditioned and non-air-conditioned buses in the incidence of higher concentrations of the studied genus of mold observed after starting of the system were not statistically significant (*p* > 0.05).

Referring now to the period of use of the system, in the air-conditioned buses, higher (by 10%) incidence of higher mold levels after a long period of system use soon after its starting was observed only for mold of the genus *Alternaria*. For the *Penicillium* mold, the incidence was at the same level, while for the *Aspergillus* and *Cladosporium* molds, the incidence was lower. In the non-air-conditioned buses after a longer period of use of the ventilation system, the incidence of higher concentration of mold after starting of the system was the same for the *Aspergillus* and *Cladosporium* molds, and lower for the *Penicillium* and *Alternaria* molds. The differences in the incidence of higher concentrations of studied mold genus after the starting of the system in both bus types for different periods of system operation were not statistically significant (*p* > 0.05).

The percentage of vehicles with higher levels of *Fumigati section* in the indoor air following starting of the system after 1 week of use was lower in air-conditioned than non-air-conditioned buses, while after 22 weeks of operation of the system in both types of buses, the percentage was at the same level equal to 0%. The observed differences in frequencies of higher levels of *Fumigati section* after starting of the system for different periods of its use, and for different types of vehicles at the same period of its use were not statistically significant (*p* > 0.05).

## Discussion

The research indicates that microclimatic conditions in the analyzed buses in the driver’s cabin before the ACS or VS were switched on largely corresponded with the conditions outside the vehicles. Therefore, it seems that the differences in average values of temperature and humidity between BACS and BVS could result mostly from the differences in values of those parameters in atmospheric background of the research. However, although the average temperatures inside the buses were similar to those outside, yet the average relative humidity of indoor air was almost always higher than that of the outdoor air. This may point to a source of dampness inside the investigated vehicles or to its excessive accumulation in result of poor exchange of the air. In some buses the relative humidity of indoor air was over 70%, which could cause condensation of steam on cold internal surfaces of the vehicle, such as upholstery, window seals etc., and thereby create good conditions for growth of mold (Piecková 2012).

Analyzing the results of the study should take into account that the level of fungal contamination is probably underestimated due to only culture based-methods use. According to the literature fungal identification should be assessed by using of molecular methods (DNA sequence analysis) or proteome fingerprint analysis (matrix-assisted laser desorption/ionization time-of-flight mass spectrometry, MALDI-TOF MS (Gautier et al. 2016) to ensure the whole picture of fungal species spectrum present in the environment. The second important issue is that in the air are present viable and non-viable microorganisms and both of them can be harmful for exposed people. Therefore the use of only culture based-methods could be considered as important limitation of the study.

To assess the fungal air contamination are recommended sampling made with using active method (volumetric air samplers), passive methods (sedimentation on the plate with nutrition media), and analyses of samples of settled dust as a carrier of microorganisms (samples of settled dust took with vacuum cleaner, electrostatic tools, print plates, and swabs). The type of method should be pointed depending on route of exposure (Leppänen et al. 2017). The drivers of buses are exposed to fungi mainly by inhalation, thus the fungal exposure in our study was assessed by active (impact) method.

The concentration of mold present in indoor air of both types of buses before switching on ACS or VS is lower than outside, which indicates the lack of indoor source of fungal microflora. The same correlation was found by Lee et al. (2006) in a similar study related to flats. At the same time the differences which we observed in our research in indoor concentrations of mold between air-conditioned buses and the buses without air-conditioning were similar to the differences between their relevant levels of mold in outdoor background. This may be explained by observation made by Dassonville et al. (2008) which revealed that atmospheric levels might largely affect the concentrations of mold indoors. The observed levels of mold in the air of air-conditioned buses and in external background in this study were higher than those obtained respectively in the study carried out by Wang et al. (2013). When the air-conditioning or ventilation system was switched on, the air contamination with fungal microflora was decreased by 68–78%; decrease having been somewhat lower in air-conditioned buses. A lower level of fungi after the air-conditioning or ventilation was turned on was also observed in the study carried out by Wang et al. (2013), but they found a more pronounced decrease for cars with turned on air-conditioning. While Vonberg et al. (2010) showed an average decrease of fungal spores in cars after air-conditioning at the 83.3% level was turned on in the vehicles. In some cases in the presented study by us, even a 78% increase in mold concentration was observed after ACS had been switched on. It may indicate that the ACS or cabin filter is contaminated with this microflora, and a longer use of this system contributed to a higher increase in mold concentration inside the driver’s cabin in BACS.

The tested buses contained allergenic molds of genera: *Aspergillus*, *Penicillium*, *Cladosporium*, *and Alternaria* as well as *Fumigati* section. The same genera of fungi were observed in vehicles indoor air by Jo and Lee (2008). No matter how long the air-conditioning or ventilation systems had been used, before they were switched on, the predominant molds in indoor air of both types of the buses were those of *Cladosporium* genus, similarly to the research carried out by Wang et al. (2013). In air-conditioned buses the concentrations of molds of *Aspergillus and Penicillium* genera were higher, which may indicate the presence of an internal source of these fungi. Once the system was switched on, no matter how long it was used, a lower average concentration of all tested genera of indoor air molds was found in both types of buses. With exception for BVS checked after 1 week of using the system, where a slight increase in average concentration of *Alternaria* mold was found. This observation was confirmed by the publication of Vonberg et al. (2010) about the air-conditioning system reducing the fungal microflora. Considering the system operation period, after 22 weeks of its use in both types of the vehicles a lower (compared to that after 1 week of operation) decrease was found in the average concentration of *Penicillium* genus mold. In the case of mold of *Alternaria* and *Cladosporium* genera, such correlation was found only for BACS. This may point to the air conditioning system’s lower capability to purify the air after a longer operation.

In the indoor air of some of the investigated vehicles the *Fumigati section* was found before the air-conditioning or ventilation system was switched on. Yet, the average indoor concentration of this species before ACS was switched on had been higher than that in atmospheric air where it was not found. This may prove the existence of an indoor source of this mold in the investigated buses. Once the system was switched on, no matter how long it was operated the average concentration of *Fumigati section* was decreased in indoor air of both types of buses.

The analysis showed for both types of the tested vehicles a low incidence (0–25%) of the occurrence of an increased concentration of both types of mold after the system was switched on, no matter how long it was used. In BACS a higher rate of higher concentration of *Cladosporium* mold was shown after the system had been switched on—no matter how long it was used. Taking into consideration the system’s operation period, in BACS after a longer period of its use only for *Alternaria* genus an increase (by 10%) in the incidence of higher concentration was observed after the system had been switched on.

## Conclusions

Although the study has some limitations (a low number of samples, possible underestimation of airborne fungi concentration caused by using only culture based-methods and sampling made only by active method), the results lead to the following conclusions:

1. Results do not provide strong evidence that air-conditioning system is an additional source of indoor air contamination with fungi. Only in few buses was showed higher concentrations of fungi. A longer operation of the system promoted increase of fungi concentration in air-conditioned buses only.
2. After switching on the air conditioning system in air conditioned buses and ventilation system in buses without air conditioning, the air contamination with fungal microflora was decreased, and that decrease was smaller in air conditioned buses.
3. Both in the air-conditioned buses and in the buses without air conditioning system, the presence of allergic mold of genera: *Aspergillus*, *Penicillium*, *Cladosporium*, *and Alternaria* and *Fumigati* section was confirmed. The incidence of higher concentration of allergic genera of fungi and *Fumigati* section after the system of air conditioning or ventilation had been switched on was determined neither by the type of the system nor by the duration of the system operation period.
